# Supplementary figures and images for: Detection of mcr-1-1 Positive Enteropathogenic Escherichia coli Isolates Associated with Post-Weaning Diarrhoea in an Organic Piglet-Producing Farm in Austria
Source: Microorganisms. 2024 Jan 24;12(2):244. doi: 10.3390/microorganisms12020244 (PMC10893164; doi:10.3390/microorganisms12020244)

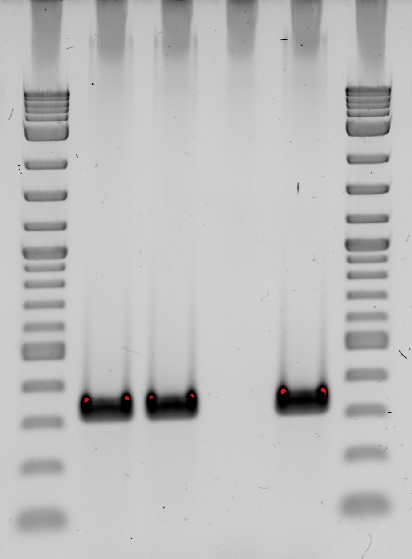

Supplement: Supplementary file 1 [file microorganisms-12-00244-s001.zip › microorganisms-2820690-supplementary.jpg]
